# Supplementary material for: Primary Cutaneous Nocardiosis (Lymphangitic Type) in an Immunocompetent Patient: A Case Report
Source: Microorganisms. 2025 Apr 29;13(5):1022. doi: 10.3390/microorganisms13051022 (PMC12114168; doi:10.3390/microorganisms13051022)
Supplement: Supplementary file 1 [file microorganisms-13-01022-s001.zip › microorganisms-3559501-supplementary.pdf]

CARE case report guidelines

CARE Checklist of information to include when writing a case report

(cc) BY-NC-ND

Topic	Item	Checklist item description	Reported on Line
Title	1	The diagnosis or intervention of primary focus followed by the words "case report"	Lines 2-3
Key Words	2	2 to 5 key words that identify diagnoses or interventions in this case report, including "case report"	Lines 42-43
Abstract (no references)	3a	Introduction: What is unique about this case and what does it add to the scientific literature?	Lines 27-41
	3b	Main symptoms and/or important clinical findings	Lines 109-126
	3c	The main diagnoses, therapeutic interventions, and outcomes	Lines 128-165
	3d	Conclusion—What is the main "take-away" lesson(s) from this case?	Lines 275-285
Introduction	4	One or two paragraphs summarizing why this case is unique (**may include references**)	Lines 46-107
Patient Information	5a	De-identified patient specific information.	Lines 109-110
	5b	Primary concerns and symptoms of the patient.	Lines 111-115
	5c	Medical, family, and psycho-social history including relevant genetic information	
	5d	Relevant past interventions with outcomes	
Clinical Findings	6	Describe significant physical examination (PE) and important clinical findings.	Lines 120-126
Timeline	7	Historical and current information from this episode of care organized as a timeline	Lines 114-115
Diagnostic Assessment	8a	Diagnostic testing (such as PE, laboratory testing, imaging, surveys).	Lines 123-126
	8b	Diagnostic challenges (such as access to testing, financial, or cultural)	
	8c	Diagnosis (including other diagnoses considered)	Lines 128-154
	8d	Prognosis (such as staging in oncology) where applicable	
Therapeutic Intervention	9a	Types of therapeutic intervention (such as pharmacologic, surgical, preventive, self-care)	Lines 155-157
	9b	Administration of therapeutic intervention (such as dosage, strength, duration)	Lines 161-165
	9c	Changes in therapeutic intervention (with rationale)	lines 157-160
Follow-up and Outcomes	10a	Clinician and patient-assessed outcomes (if available)	
	10b	Important follow-up diagnostic and other test results	Lines 157-161
	10c	Intervention adherence and tolerability (How was this assessed?)	
	10d	Adverse and unanticipated events	
Discussion	11a	A scientific discussion of the strengths AND limitations associated with this case report	Lines 267-271
	11b	Discussion of the relevant medical literature **with references**.	Lines 169-271
	11c	The scientific rationale for any conclusions (including assessment of possible causes)	
	11d	The primary "take-away" lessons of this case report (without references) in a one paragraph conclusion	Lines 275-285
Patient Perspective	12	The patient should share their perspective in one to two paragraphs on the treatment(s) they received	
Informed Consent	13	Did the patient give informed consent? Please provide if requested	**Yes** ☒ **No** ☐
